# Low-Velocity Impact Analysis of Pineapple Leaf Fiber (PALF) Hybrid Composites

**DOI:** 10.3390/polym13183194

**Published:** 2021-09-21

**Authors:** Muhammad Imran Najeeb, Mohamed Thariq Hameed Sultan, Ain Umaira Md Shah, Siti Madiha Muhammad Amir, Syafiqah Nur Azrie Safri, Mohammad Jawaid, Mohamad Rabaie Shari

**Affiliations:** 1 Department of Aerospace Engineering, Faculty of Engineering, Universiti Putra Malaysia, UPM Serdang 43400, Selangor Darul Ehsan, Malaysia; muhdimran02@gmail.com (M.I.N.); ainumaira91@gmail.com (A.U.M.S.); 2 Laboratory of Biocomposite Technology, Institute of Tropical Forestry and Forest Products (INTROP), Universiti Putra Malaysia, UPM Serdang 43400, Selangor Darul Ehsan, Malaysia; snasafri@gmail.com (S.N.A.S.); jawaid@upm.edu.my (M.J.); 3 Aerospace Malaysia Innovation Centre (944751-A), Prime Minister’s Department, MIGHT Partnership Hub, Jalan Impact, Cyberjaya 63000, Selangor Darul Ehsan, Malaysia; 4 Industrial Technology Division, Malaysian Nuclear Agency, Bangi 43000, Selangor Darul Ehsan, Malaysia; madiha_amir@nuclearmalaysia.gov.my (S.M.M.A.); rabaie@nuclearmalaysia.gov.my (M.R.S.)

**Keywords:** plant fiber, pineapple leaf fiber, PALF, low-velocity impact, computed tomography, composite, damage

## Abstract

The low-velocity impact behaviour of pineapple leaf fiber, PALF reinforce epoxy composite (P), PALF hybrid (GPG), and four-layer woven glass fiber (GGGG) composite was investigated. As for post-impact analysis, the damage evaluation was assessed through photographic images and X-ray computed tomography, using CT scan techniques. The key findings from this study are that a positive hybrid effect of PALF as a reinforcement was seen where the GPG shows the delayed time taken for damage initiation and propagation through the whole sample compared to GGGG. This clearly shows that the addition of fibers does have comparable composite properties with a fully synthetic composite. Through the visual inspection captured by photographic image, the presence of woven fiber glass mat in GPG presents a different damage mode compared to P. Moreover, CT scan results show extended internal damage at the cross-section of all impacted composite.

## 1. Introduction

Impact damage can compromise performance as well as reliability of composite materials and, therefore, it warrants serious attention. Impact damage can occur either during in-service applications or handling during the manufacturing process. As a composite is subjected to an impact, it will experience a bending stress. The bending properties of a composite can be determined through flexural test. The flexural test provides the composite bending strength and its stiffness. The flexural properties of a composite provides a preliminary insight on the possible impact strength properties of a composite. Generally, literature studies show that when the flexural strength of a hybrid composite is higher than non-hybrid, then the impact strength will have the same kind of result. A study shows that hybrid flax/carbon/epoxy composite has had an improved flexural strength of about 345.191% compared to flax/epoxy, while in impact test, flax/carbon/epoxy has 479.57% higher peak force compared with the flax/epoxy [1]. Besides that, hybrid basalt/glass/epoxy shows 30.35% higher flexural strength compared to basalt/epoxy and, in impact test, basalt/glass/epoxy shows 17.24% improvement of peak force compared to basalt/epoxy [2]. However, more comprehensive studies are needed to see a pattern between flexural and impact strength of a composite.

In low-velocity impact, the type of damage mode created depends on the impact parameters (fabrication, layout sequence, fabrication method, impactor incidence angle, and its geometry) and the properties (type of fiber, type of binder) of the composite materials [3]. One of the failure mechanisms in fiber-reinforced composite materials is presented in the form of fiber pull-out. Studies have shown that fiber pull-out can occur after subjecting composites to low-velocity impact [4]. Apart from fiber pull-out, low-velocity impact can also result in matrix cracks on the surface of a specimen. The damages originate from fiber fracture leading to bending and/or stretching, thus resulting in fiber pull-out and matrix cracks. Studies have also shown that laminated composite experience predominant delamination damage mode because of inter-layer debonding [4]. Vertical displacement of broken and unbroken fibers result in delamination. Different studies have shown that delamination occurs within interior interfaces due to different fiber orientations of adjacent plies [3].

The impact resistance of composites can be further improved via hybridisation, as it offers a wide range of mechanical properties that cannot be achieved by using a single type of fiber. The impact performance can be evaluated in terms of its peak force and absorbed energy. The peak force represents the maximum load that a laminate composite could bear upon impact before experiencing major damage, whereas the absorbed energy reflects the unrecoverable dissipated energy within the system. A study on impact resistance of bamboo/glass hybrid composite with and without 0.5% wt of CNT had shown that the presence of filler does help improve impact resistance, with up to 36.23% increase in peak force [5]. Results also showed that flax/basalt hybrid displayed higher peak force by approximately 50% compared with flax composite when subjected to varying impact energy of 50 J, 60 J, and 70 J [6].

Existing damage detection methods hinge on various principles, thus displaying a range of results regardless of the visual damage. Non-destructive testing (NDT) is among the various methods available to evaluate damage to materials or components. NDT involves the identification and characterization of damage on the surface as well as to the internal structure without the need to cut or alter the materials [7]. Hence the NDT technique is vital in evaluating the structural sustainability of a material without damaging its components.

X-ray computed tomography, or CT, is one of the NDT techniques that can be applied to evaluate damage. Unlike ultrasonics, CT cannot capture the volumetric characteristics of defects. However, the CT technique can be applied to gauge the planar aspects of defects at a higher resolution. Hence, CT is a powerful technique used to check the presence of major defects, such as open cracks and porosity. Besides those defects, a different study shows that micro-CT testing are used to capture the composite debonding length reduction of a surface modification of flax yarns by enzymatic treatment [8]. Moreover, through X-ray micro computed tomography also can check fiber distribution throughout a composite. A study on internal structure of sisal/kenaf composite with bagasse ash (BGA) as a filler captures only a few filler agglomerations [9]. In addition, a study on influence of stack sequence of UHMWPE (P), flax (F) and jute (J) hybrid composite subjected to flexural loading was investigated [10]. Through computed tomography test, it showed that FF-J-PP-J-FF composite had extensive fiber breakage followed by delamination of the composite specimen, with some areas of the specimen being intact. On the other hand, JJ-P-FF-P-JJ composite showed shearing and delamination of laminates. Next, CT images can also capture the presence of voids and fiber orientation. A study was conducted of the x–z section of the 90°-oriented fiber composite with/without tensile load [11]. No void generation or growth was observed. Furthermore, it also reported that the CT image of the longitudinal cross-section of a fiber particle is seen in the x–z planar image. The fiber was oriented in the z-axis direction. The fibers present as brighter phases than the matrix. Further, studies on 2D and 3D woven composite subjected to LVI test showed that the area of damage in 3D woven composite was relatively smaller compared to 2D woven composite, as captured in X-ray CT. This is because the binding yarns in 3D woven composite suppress the interlaminar damages from propagating across the plies [12].

Therefore, in this paper, in-depth assessment of low-velocity impact composite behaviour properties and post-damage analysis on the impacted composites is carried out using the radiography NDT method. Multi-angled analysis is needed to fully understand the behaviour of composites post impact. In this novel study, pineapple leaf fiber (PALF) from Yankee’s pineapple crop variant was used as a composite reinforcement because of its high-tensile strength properties and it had a rough surface morphology [13]. Flexural, low-velocity impact (LVI) and CT scan tests are carried out to ascertain the properties of the composite. These tests are critical to evaluate the composite structural integrity towards lightweight interior vehicle components. With that, the use of PALF composite in automobile interior component application can therefore be tapped in view of its great potential.

## 2. Materials and Methods

### 2.1. Preparation of Materials

The natural fiber used in this research was Yankee variant pineapple leaves fiber (PALF) extracted from one- to two-year-old crop grown in Teluk Panglima Garang in Selangor, Malaysia. The fiber extraction process was prepared according to Najeeb et al. (2020) [13]. Analysis of the chemical composition of PALF showed a high content of cellulose (47.74%), followed by hemicellulose (15.98%), and lignin (2.44%) [14]. A glass fiber mat E600 purchased from Mechasolve Engineering Sendirian Berhad (Selangor, Malaysia) was used to fabricate the hybrid, as well as for the glass fiber composite. The density, tensile strength, and tensile modulus properties of the glass fiber are 2.58 g/cm^3^, 3445 MPa, and 72.3 GPa, respectively [15]. Both Smooth-On brand epoxy and hardener (Pennsylvania, USA) are used. The type of epoxy used is Epoxamite100 and 103 slow hardener. The density, tensile strength, and tensile modulus properties of epoxy are 1.10 g/cm^3^, 54 MPa, 3.2 GPa, respectively [15].

### 2.2. Composites Preparation

**Figure 1:**
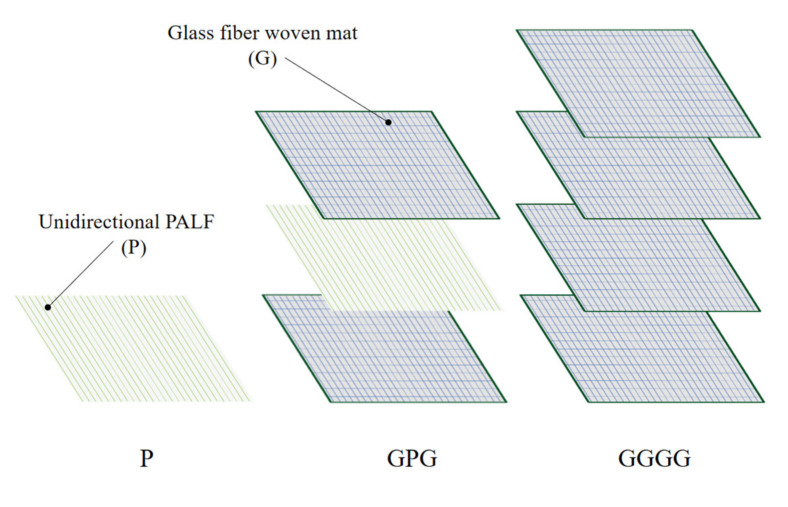
Schematic of fiber composition layup.

The PALF/epoxy composites were produced by aligning the PALF in unidirectional (UD). Thermoset composites epoxy were used because of their favorable properties, such as good chemical resistance, light weight, good mechanical strength, and high glass transition temperature [16]. The loading of PALF was fixed at 10% by weight, and the thickness of composite produced was 3 mm. The respective PALF loading was chosen based on previous studies affirming its high mechanical properties [17]. As for the hybrid composite, the weight of PALF is 5% wt because it has glass fiber mat on the top and bottom layers of the composite. Reddy et al. (2021) shows that this layering sequence has the highest strength and as for synthetic composite, the layering is made up to four layers so that the fabricated composite is 3 mm which is constant with all fabricated composites [18]. Those composites manufactured for the experiment are described in Table 1 and the schematic of the fiber composition is presented in Figure 1. The composites were prepared using the hand layup method. Before starting the fabrication process, the surface of the mold was waxed for ease of removal. The ratio used for epoxy to hardener was 3:1 and the mixture was stirred uniformly, and was then used to impregnate with the fiber in a mold. The curing process of the composite is at room temperature for 24 h.

### 2.3. Flexural Test

The flexural strength properties test was carried out using INSTRON 5567 (Institute of Tropical Forestry and Forest Products (INTROP)’s Biocomposite Laboratory, Universiti Putra Malaysia, Malaysia). ASTM D7264 standard was applied to carry out the three-point bending test on the composite, with span to thickness ratio of 32:1, and width of 13 mm. The crosshead speed was constant at 2 mm/min.

### 2.4. Low-Velocity Test

The Drop Weight Impact Tester (Imatek IM10, Department of Aerospace Engineering, Universiti Putra Malaysia, Malaysia) was used to test the impact strength of the composite. The impact energy ranged between 1.0–12.0 J. The impact energy E was calculated using Equation (1).

E = mgh(1)

where E represents energy (J), m represents mass (kg), g represents the gravitational acceleration (ms^−2^), and h represents the height of impactor (m). The output from these tests were energy, load, and displacement as a function of time. The low-velocity impact is conducted according to ASTM D7136. The damage was subjected throughout-of-plane, concentrated impact (perpendicular to the plane of the center composite plate) using a drop weight with a hemispherical striker tip. The dimension of the samples is 10 cm × 15 cm × 0.3 cm. The PALF weighted for P composite was 3 g and in GPG was 1.5 g. The total mass and volume fiber content in GPG are 17.5 g and 7.5 cm^3^, respectively. Moreover, the total mass and volume fiber content in GGGG are 32 g and 12 cm^3^, respectively.

### 2.5. Non-Destructive Test

**Figure 2:**
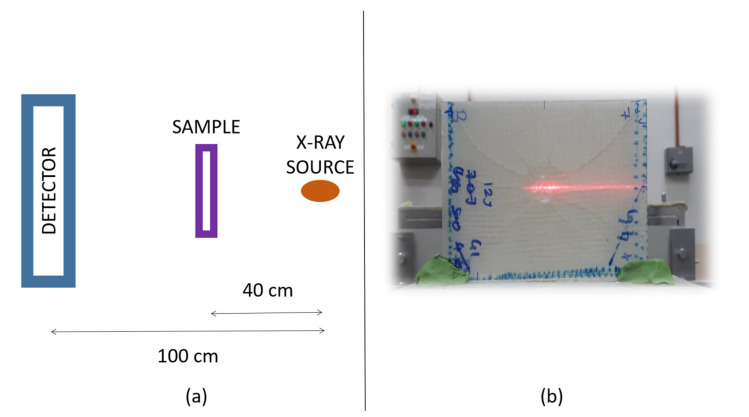
(**a**) The distance between X-ray source and the detector; (**b**) horizontal sample setup.

The low-impacted damage mode on composite was evaluated using X-ray computed tomography. The instrument used was a YXLON (Germany), MGC 41, and 160 kV. The X-ray was set at 30 kV and 2 mA, using linear array detector. The distance between the X-ray radiation source and the sample was set at 40 cm; and distance between the source and detector was fixed at 100 cm as shown in Figure 2a. The sample was placed horizontally, and scanning was carried out at the center of the composite as shown in Figure 2b.

## 3. Results

### 3.1. Flexural Test

**Figure 3:**
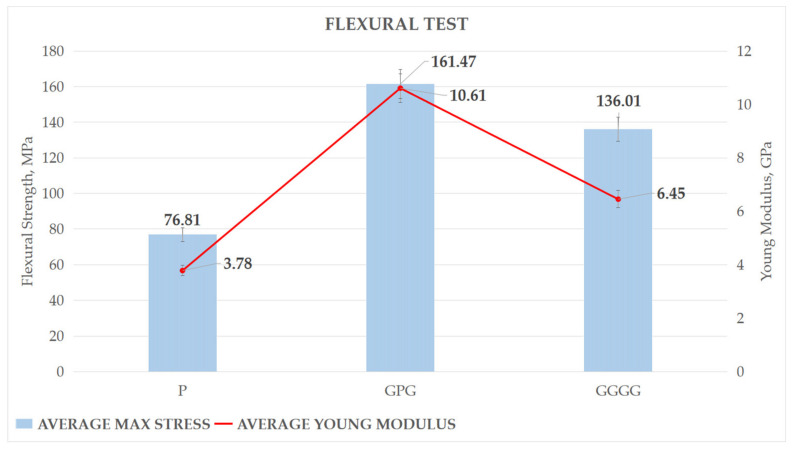
Flexural strength and young modulus of composites.

**Figure 4:**
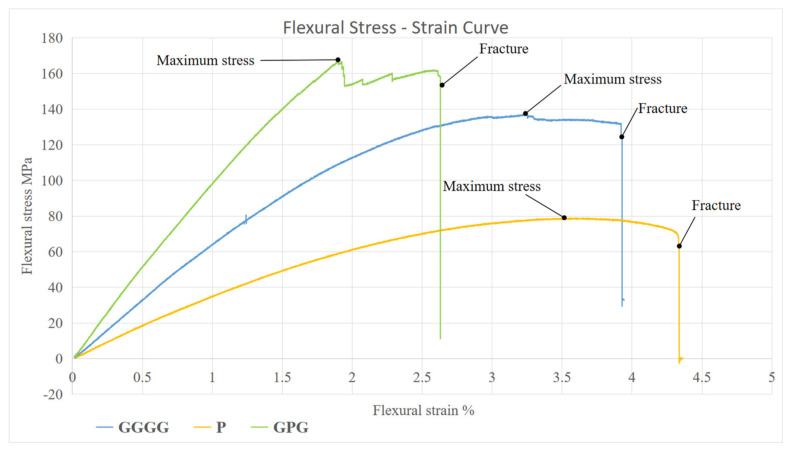
Stress-strain curve of composites.

Flexural strength results of all three composites are presented in Figure 3. Figure 3 shows that P had the lowest flexural strength about 76.81 MPa. A positive impact of hybridization shows that the highest flexural strength was shown by GPG composite, 161.47 MPa, which is about 18.72% more than GGGG. Besides GPG having the highest flexural strength, it also shows highest stiffness, where the young modulus shows 10.61 GPa, followed by GGGG and P, 6.45 GPa, and 3.78 GPa, respectively. This means the GPG has the smallest elastic deformation for a given applied load followed by GGGG and P. Permanent deformation represents material failure. The findings were further supported by literature where hybridized fiber glass with kenaf fiber composite yields 142% more flexural strength compared to kenaf composite [19]. Besides that, studies on banana fiber (B)/glass fiber hybrid with stacking sequence G/B/G shows that hybrid banana poses higher flexural properties compared to banana epoxy composite which is 78.7 MPa and 46.04 MPa, respectively [18]. Studies show that plant fiber has good bonding between the fibers and matrix that evince the improvement in the flexural properties of the composite [20,21]. The findings were supported by the scanning electron microscope, using SEM morphology analysis, which showed the intimate contact between the fiber and matrix [21]. This results in slowing down the crack propagation between the layers as the load stress is distributed uniformly throughout the composite by the fibers and matrix. Apart from that, such properties were achieved in hybrid composite because the composite properties are mainly dependent on the modulus and the elongation at break of the individual reinforcing fibres [22]. E-glass fiber display 73 GPa of young modulus and 4.8% of elongation at failure, while most plant leaf fiber had 20–40 GPa of young modulus and 1.5–7% elongation at failure [23]. Moreover, Figure 4 shows the stress–strain, or SS, curve of the composite. It shows that GPG, after peaking at maximum stress, has a sudden drop in stress, indicating that the sample is cracked. However, with the presence of PALF as the internal layer in the hybrid composite, GPG helps to extend the composite bending performance before it reaches a fracture stage. This shows that the presence of PALF broadens the plastic region of the composite, as shown by P–SS curve, where P physical deformation up to 4.4%, compared to GGGG which has 3.9% only. The natural fiber PALF is better at distributing the load between fiber and matrix compared to glass fiber.

### 3.2. Low-Velocity Impact Test

**Figure 5:**
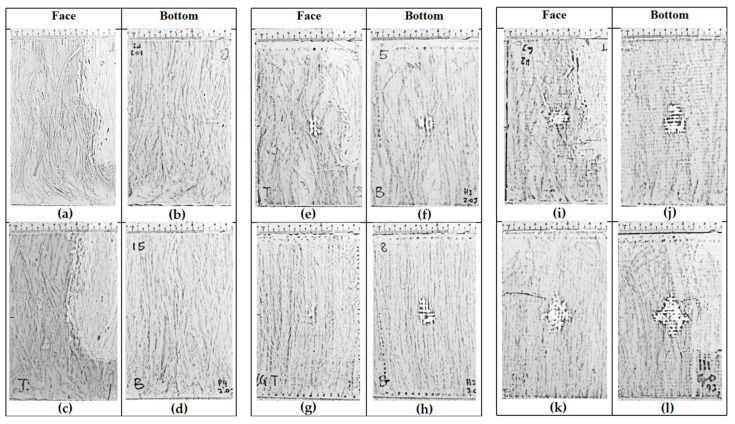
Schematic diagram of the P and GPG impacted samples according to the energy level: (**a**) P 1.0 J, (**b**) P 1.0 J, (**c**) P 2.0 J, (**d**) P 2.0 J, (**e**) GPG 2.0 J, (**f**) GPG 2.0 J, (**g**) GPG 3.0 J, (**h**) GPG 3.0 J, (**i**) GPG 6.0 J, (**j**) GPG 6.0 J, (**k**) GPG 9.0 J and (**l**) GPG 9.0 J.

**Figure 6:**
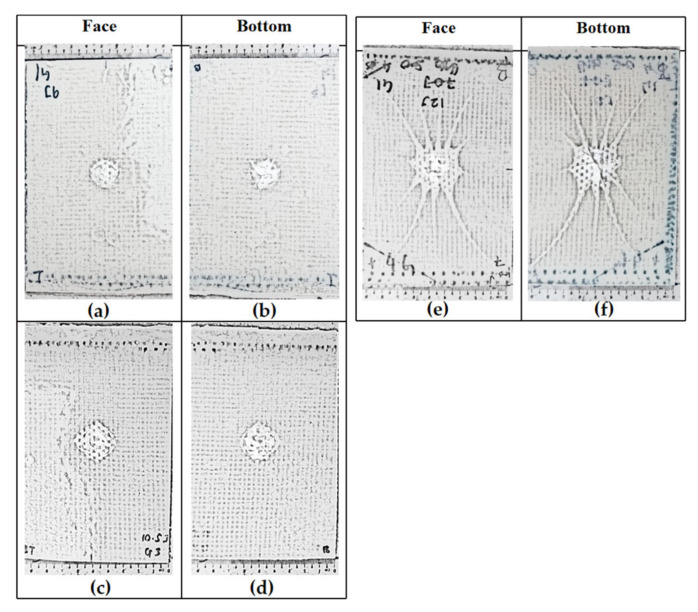
Schematic diagram of the GGGG impacted samples according to the energy level: (**a**) GGGG 9.0 J, (**b**) GGGG 9.0 J, (**c**) GGGG 10.5 J, (**d**) GGGG 10.5 J, (**e**) GGGG 12.0 J and (**f**) GGGG 12.0 J.

An impact test was carried out after ascertaining the strength of each composite based flexural property. Bensadoun et al. (2017) reported the relation between flexural test with impact test [24]. In his study, the author found that the composite with high flexural strength displays high peak-impact force, where strong material will sustain more load under impact before it fractures, leading to an increase in energy absorption. From Section 3.1, it was ascertained that GPG and GGGG possess higher flexural strength and stiffness compared to P. The flexural test gave the author the early idea to set up the impact energy range for each composite. The aim of this impact study is to observe the impact damage characteristic up to its prior perforation (before total failure) for each composite. Therefore, it is not necessary to begin the testing for GPG and GGGG at lower energy because the damage is not significant. The impact energy range for respected composite is presented in Table 2. Figure 5 and Figure 6 show the schematic diagram of P. GPG and GGGG impacted composite.

#### 3.2.1. Force–Displacement Curve

**Figure 7:**
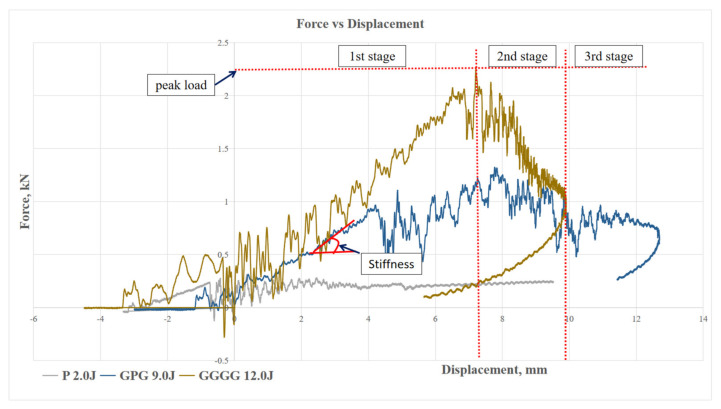
Force–Displacement curve of maximum impact energy prior to composites perforation.

**Figure 8:**
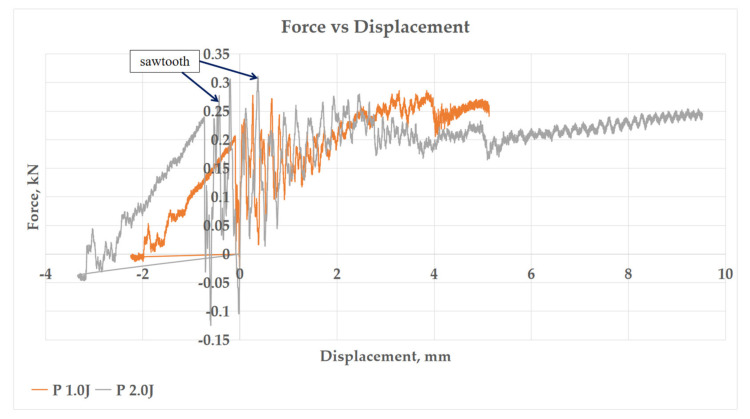
Force–Displacement curve of impact energy prior to P perforation.

**Figure 9:**
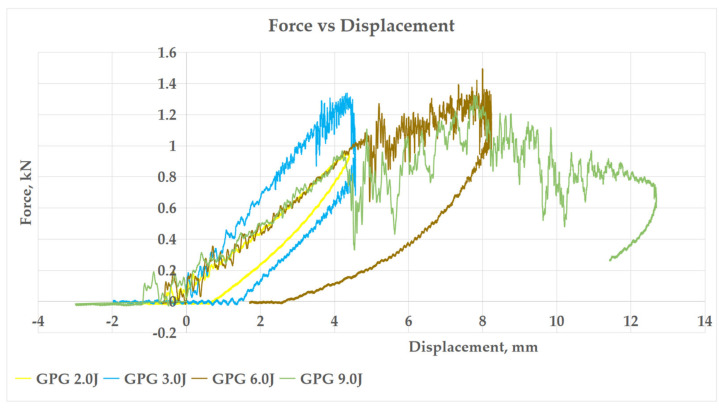
Force–Displacement curve of impact energy prior to GPG perforation.

**Figure 10:**
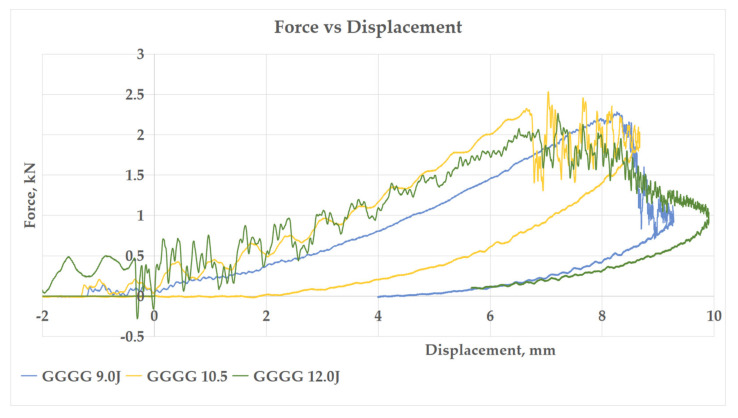
Force–Displacement curve of impact energy prior to GGGG perforation.

Figure 7, Figure 8, Figure 9 and Figure 10 show the impact force–displacement curve of three composite samples, namely P, GPG, and GGGG, at various energy levels. The oscillations observed on the force–displacement curves are associated with the natural modes of vibration of the impacting system (shaft, hammer, and impact sensor) [25]. The P was subjected to impact energy of 1 J and 2 J. The P experienced perforation when subjected to impact energy above 2 J. Therefore, in this study the maximum impact energy for P was limited to 2 J. The experiment showed that the GPG can withstand up to 9 J impact energy, which is 350% higher than P, before experiencing perforation. Meanwhile, the GGGG composite can withstand a maximum impact energy of 12 J, which is 33.33% higher than GPG, before experiencing perforation.

As shown in Figure 7, P shows the lowest slope compared to GPG and GGGG composite in the first stage of the graph. This indicates that P has the lowest stiffness property. This finding is also in line with flexural stiffness portrayed by P. This means that P is most brittle compared to GPG and GGGG composites. This finding was further supported by the open loop P graph (refer to Figure 8), indicating that the composite did not fully take the rebound during impact, showing that the composite has less elasticity properties [26]. The P composite had the same force displacement graph pattern with kenaf/epoxy composite reported by Majid et al. (2018) where, at stage 1, the loading is proportional with displacement until a “sawtooth” curve formed at peak force before unloading takes place [27]. Such a curve characteristic was presented due to the vibrational motion between the impactor and the composite.

In contrast, both the GPG and GGGG composites graphs shows a close loop started with the loading curve increasing proportionally until it reached a peak force then curve returned to its initial point as shown in graph, as in Figure 9 and Figure 10. The curve shows that the composites fully took the rebound upon impact. This curve can be broken down into three stages, which are first stage linear force increment, second stage peak force, and third stage sudden force decrements. The GPG and GGGG graph shows that as the impact energy increases, the larger the area under the curve is covered. The area under the curve is the deformation energy that is initially transferred from the impactor to the composite and then returned (rebound) from the composite to the impactor [28]. This process is called the energy recovered process. At lower impact energy, 2.0–6.0 J, the GPG curve shows a sharp impact force drop after reaching the peak. This pattern was seen for GGGG at 9.0 J as well. However, at high-impact energy, 9.0 J GPG and 10.5–12.0 J GGGG as the first peak force was reached (first stage), there was a sharp drop then the curve continued to elongate and increase in impact force because of the composite resistance to prevent the striker perforating the composites [29]. At the third stage it terminated the curve by decreasing the displacement. The same trend of fluctuation after the peak point also was observed in other related research work in which the impact force remains increasing with the presence of significant oscillations in the data from the activation of two different damage mechanisms: shear damage followed by the delamination between the mat and roving layers with increasing load [26].

#### 3.2.2. Peak Force of Composite

**Figure 11:**
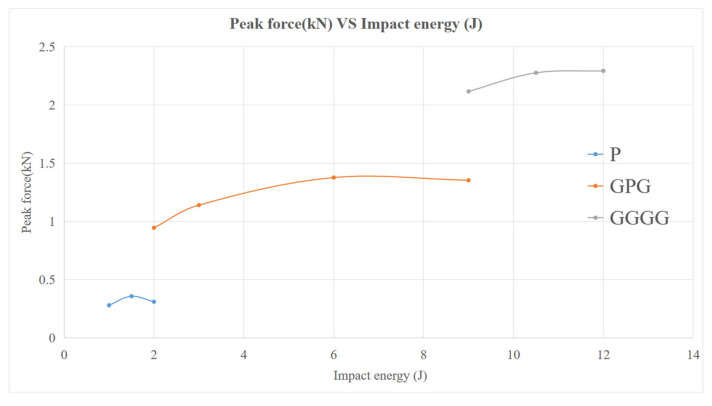
Peak force of composites.

Peak force, indicating the maximum load that a composite can withstand prior to experiencing failure during impact, is shown in Figure 11. The maximum peak force of P was recorded at 0.35 kN compared to GPG 1.37 kN, showcasing a 291.43% improvement in impact resistance. The same case was also observed in Majid et al. (2018). At maximum-impact energy kenaf hybrid with glass fiber shows an increment in peak force compared to kenaf composite from approximately 0.9 kN to 1.5 kN [27]. This is because the glass fiber helps to improve damage impact-resistance by reducing the damage propagation and localizing the damage at the point of impact [30]. The maximum peak force of GGGG was 2.29 kN, which is 67.15% higher than the GPG. Even though the GPG contains only two layers of glass fiber-woven mat, the added PALF fiber plays an important role in taking the impact and distributing it throughout the matrix. Furthermore, the presence of PALF had improved the composite impact-resistance capacity by increasing the impact time as shown in the impact vs. time curve. The shift of the curve peak point towards the right-hand side shows the longer period for achieving its maximum peak [27].

Moreover, the trend of the three composites shows decreased peak force at each respective maximum-impact peak force. This indicates that at the highest impact energy that each composite can bear, the composite experiences a drop in its flexibility (due to major damage) to absorb the impact force [31].

#### 3.2.3. Energy–Time Curve

**Figure 12:**
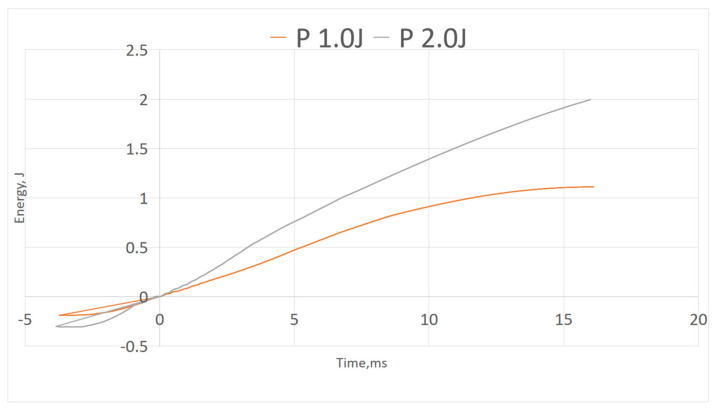
Energy-time of P.

**Figure 13:**
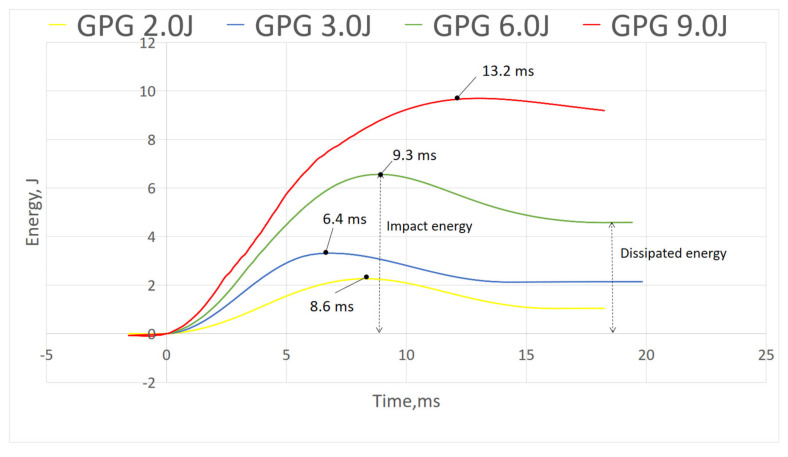
Energy-time of GPG.

**Figure 14:**
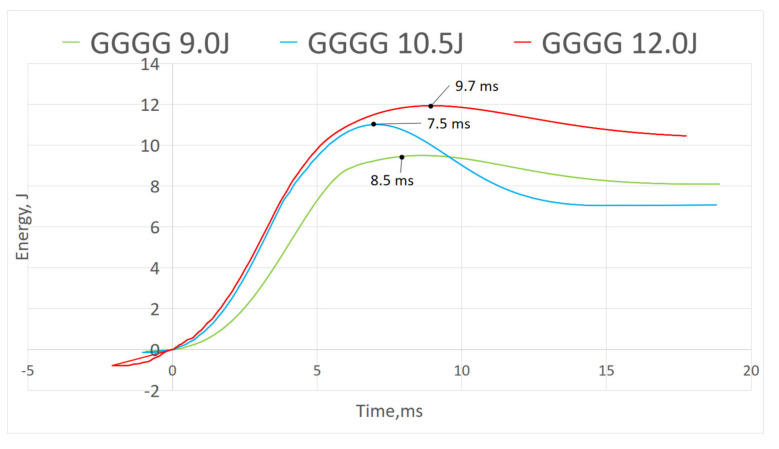
Energy-time of GGGG.

Figure 12, Figure 13 and Figure 14 show energy–time history of three composites under different impact energy, obtained by software. Figure 12 shows P did not display curve peak, indicating the composite did not experience rebound upon impact as shown in force–displacement curve where there was no close loop presented. Whereas in both Figure 13 and Figure 14, GPG and GGGG composites displayed the maximum energy peak before dropping into constant energy; the maximum peak presenting the impact energy while the constant energy represents the energy dissipated by the samples [25]. GPG displayed longer time to reach peak compared to GGGG which is on average 9.4 ms and 8.6 ms, respectively. This shows that the GPG layering had slowed down the time taken for damage initiation and propagation through the whole sample [32].

### 3.3. Visual Inspection

**Figure 15:**
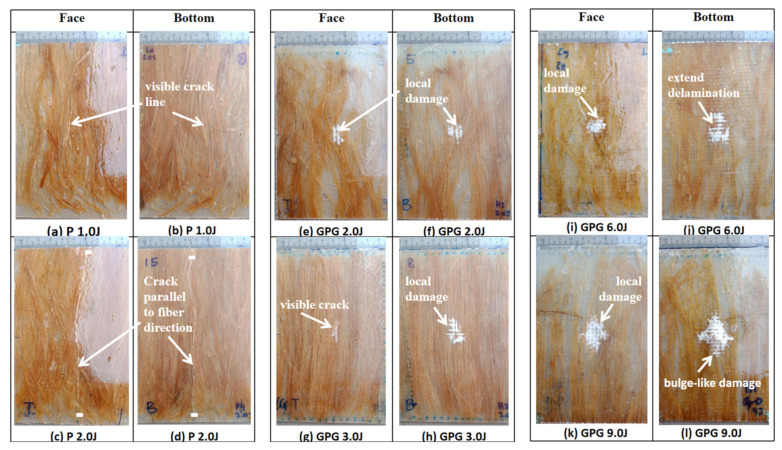
Visual damage of impacted P and GPG.

Figure 15 shows the front and back impact surface images after the impact test of the specimens. The P composite has been tested with two different measures of impact energy, 1.0 J and 2.0 J, where at 2.0 J impact energy is a prior perforation impact energy. Figure 15a–d observes that at both measures of impact energy, 1.0 J and 2.0 J, the composite was associated with a particular damage mechanism that does not give rise to the classical perforation but to a typical “transverse” failure with fracture surface parallel to the fiber direction. At 1.0 J impact energy, the crack was measured at 1.0 cm while at 2.0 J impact energy it was measured at 1.3 cm. Note the study conducted by Militello et al. (2020) which stated that the fracture mode was due to matrix crack and fiber breakage [33]. Based on the damage in Figure 15a–d show that the fiber unidirectional alignment cannot help much in resisting the load effectively through the matrix.

The impact test was carried out on the hybrid composite, GPG comprising two pieces of glass-woven mat sandwiched between 5% wt PALF placed in a unidirectional position. The impact energy subjected on the GPG composite began from 2.0 J, as it is the highest impact energy where the P composite can resist prior to perforation. The impact energy subjected on the GPG composite was set at 2.0 J, 3.0 J, 6.0 J, and 9.0 J. Unlike the P composite, the GPG composite displayed damage concentrated around the point of impact, as shown in Figure 15e–l. This is because the presence of fiber glass-woven mat can help localize the impact damage, preventing it from extensively spreading throughout the composite. A similar trend has been seen on a sandwich composite, whose top and bottom surfaces used woven glass fiber mat. Through visual inspection, it can be observed that the fibre pull-out localized at the impact contact area [34].

**Figure 16:**
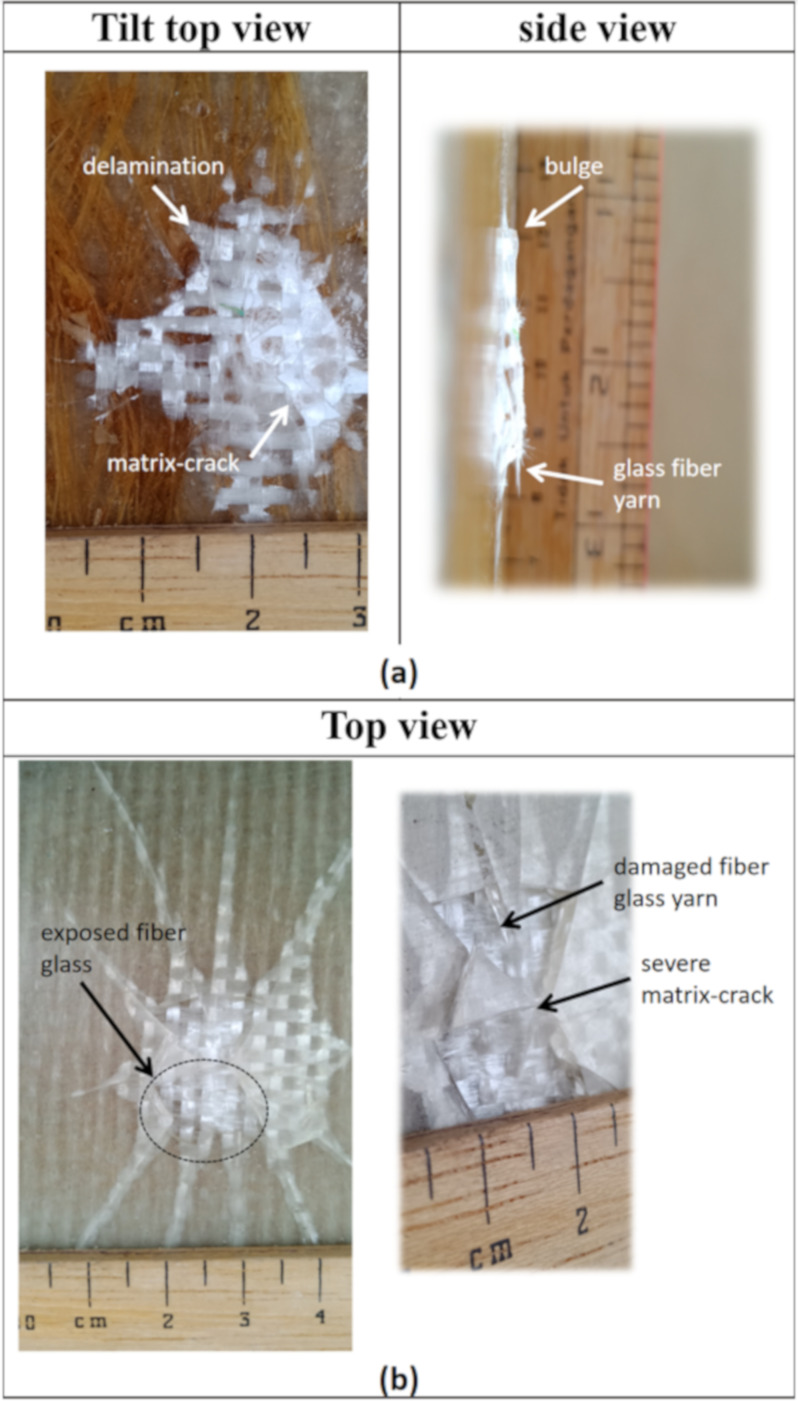
Close-up view of impacted composites: (**a**) GPG 9.0 J, (**b**) GGGG 12.0 J.

The results show the damaged area is increased when the composite is treated to higher impact energy. It was also observed that the size of the damage is similar at both the top and bottom surfaces when subjected to 2 J and 3 J impact energy. However, when subjected to impact energy of 6.0 J and 9.0 J, the size of the damaged portion at the bottom surface of the composite is doubled compared to the impacted surface at the front. This suggests that at a higher impact energy, the lowest part of the composite experienced greater tension thus creating a wider crack, compared to the upper surface which experienced compression upon impact. When subjected to 9.0 J impact energy, the bottom surface of the composite displayed greater damage due to further delamination of the glass-woven fiber mat and matrix crack as shown in Figure 16a. From the side view in Figure 16a, bulging damage can be observed after the composite is subjected to 9 J impact energy. This further supports earlier discussions pertaining to various modes of damage, including matrix crack, delamination, and fiber breakage. Beyond 9 J impact energy, the composite displayed perforation. Table 3 summarizes the typical damage mode associated with low-velocity impact for natural fiber and its hybrid composite.

**Figure 17:**
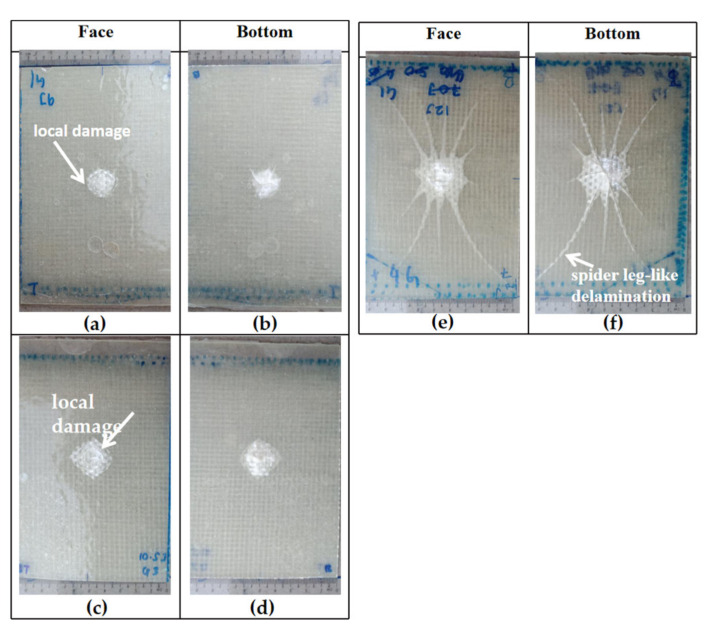
Visual damage of impacted GGGG: (**a**) GGGG 9.0 J, (**b**) GGGG 9.0 J, (**c**) GGGG 10.5 J, (**d**) GGGG 10.5 J, (**e**) GGGG 12.0 J and (**f**) GGGG 12.0 J.

The impact test was carried out on a composite made up of four layers of glass-woven mat, GGGG. As in all other composites tested in this experiment, the thickness of the GGGG was also fixed at 3 mm, in keeping with the desired application. The GGGG was subjected to impact energy of 9.0 J, 10.5 J, and 12 J. Beyond 12 J impact energy, the GGGG experienced perforation. A similar mode of damages were observed at the front and bottom of the composite upon being subjected to various impact energy up to 12 J. Damages in the form of bulge can be observed at the bottom surface of the GGGG. However, the degree of damage was relatively less severe compared to GPG. Localized circular damage can be observed on the top and bottom surfaces when the composite samples were subjected to 9 J and 10.5 J impact energy, as shown in Figure 17.

However, damages appearing like “spider legs” can be observed on both sides of the sample surface when subjected to 12 J impact energy. This shows that the composite experienced extensive delamination across the composite plate. Figure 16b shows a close-up view at the bottom surface showing that the glass-woven mat did not experience visible fiber breakage but the epoxy was severely damaged at the centre, with the appearance of broken pieces that were detached from the main structure.

### 3.4. Cross-Section Damage Investigation by CT Scan Technique

**Figure 18:**
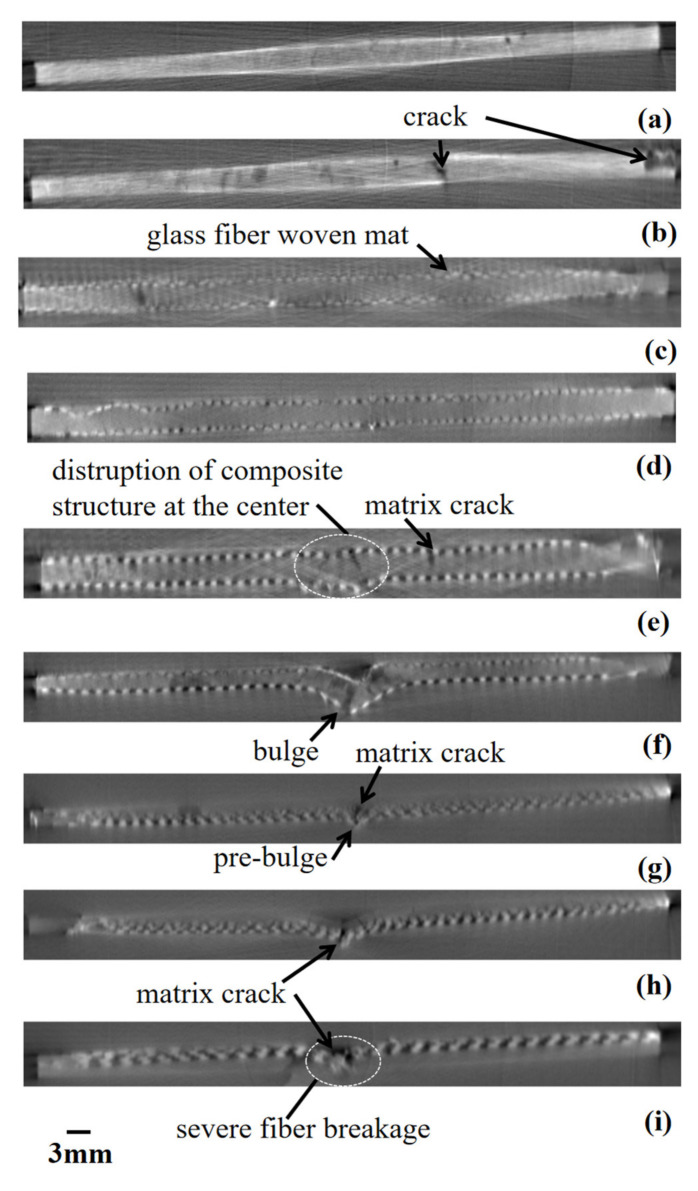
CT scan of composite horizontal cross-section: (**a**) P 1.0 J, (**b**) P 2.0 J, (**c**) GPG 2.0 J, (**d**) GPG 3.0 J, (**e**) GPG 6.0 J, (**f**) GPG 9.0 J, (**g**) GGGG 9.0 J, (**h**) GGGG 10.5 J and (**i**) GGGG 12.0 J.

Visual inspections, as captured in Figure 15, Figure 16 and Figure 17, cannot be relied upon to gauge the actual extend of the damages. Furthermore, there is still room for improvement in assessing the complexity of damage mechanisms induced by low-velocity impact energy. It is therefore vital to assess internal damage of the composites subjected to low-velocity impact. In this section, the CT scan was used to analyse the cross-section of the composite across the centre of the damaged area, as shown in Figure 18. Octopus software was used to reconstruct the sinogram image obtained from the CT scan.

No damage was detected in the P subjected to 1.0 J impact energy from the images obtained. The CT scan could not capture the line crack because the X-ray was projected at the middle of the sample which was parallel with the crack formation, as seen visually in Figure 15a–d. Damage was observed at several parts along the cross-section when the composite was subjected to 2.0 J impact energy, as seen in Figure 18b. The cross-section image showed two matric crack localities near the centre of impact and at the edge of the composite. The edge matrix crack was due to the resin-rich region as seen visually in Figure 15c. Ullah et al. (2018) reported that the matrix cracking of the composite was dominant in the resin-dominated regions, as captured by tomographic 3D images [37].

Figure 18c–f shows a dotted line-like which represents a layer of glass-woven mat at top and another layer at the bottom of the composite, with 5% wt PALF in between. The GPG did not showcase internal damage at 2 J and 3 J, as shown in Figure 18c,d. This shows that the glass-woven fiber mat can effectively absorb the impact at the lower range of low-velocity impact energy. However, at 6.0 J impact energy, as shown in Figure 18e, a disruption of fiber mat layering at the center can be seen due to the compression stress propagated through the layers [38]. Moreover, clear internal damage can be observed when subjected to 9 J impact, as in Figure 18f. It shows that the glass-woven fiber mat sustained damage at the top surface, while the glass-woven fiber mat as well as matrix showed signs of breakage at the bottom surface. A similar trend was observed by Chew et al. (2020) where the CT scan captures large matrix splits at the back surface of the composite compared to the face [36]. This was due to the development of fiber damage through the composite thickness.

The glass composite is made up of four layers of glass-woven fiber mat GGGG. It sustained localized damage at the centre when subjected to low-velocity impact energy, similar to the trend observed in the GPG. Generally, the higher the impact energy, the greater the damage caused, as seen in the cross-section of the composite. When subjected to 9 J impact energy, the damage was only observed at the upper surface of the composite with pre bulge-like shape at the bottom, as in Figure 18g. When subjected to 10.5 J impact energy, bulges appeared at the bottom surface of the composite and also glass woven mat fiber breakage can be seen. The bulge and fiber breakages appear at the bottom because of the stress waves propagated upward along the laminates due to the bending stress. The damage pattern was observed in glass fiber reinforced composite where the fiber on the impact face had inward local deformation and bending delamination was seen at the bottom part of the composite through CT scans, as reported by Zhang et al. (2018) [38]. Figure 18i shows when the impact energy is increased to 12 J, the front surface showed a dent with severe cracks appearing at the center and continuing to the bottom part of the composite. This shows that the internal cracks in the bottom grew upwards, while surface cracks grew downwards leading to the propagation of continuous cracks.

## 4. Conclusions

The low-velocity impact behaviour of pineapple leaf fiber (PALF) reinforced epoxy composite, P, PALF Hybrid, GPG, and four-layer woven glass fiber composite, GGGG was investigated. The impact performance of the composites was analyzed in terms of peak force and absorbed energy. The damage evaluation was assessed through photographic images and CT scan techniques. Through cross-section investigation by CT scan technique, it was shown that GPG and GGGG had much clearer crack damage. P did not capture much internal crack because the X-ray was projected at the middle of the sample which was parallel with the crack formation. Besides that, the CT scan also captures the GPG and GGGG layering disruption at the point of impact. This shows that, in this case, CT scan was good in capturing woven mat layering formation/deformation compared to single-strand fiber (shown by P).

In conclusion, the low-velocity impact property for PALF composite can be improved by the interlayer hybrid structure. The addition of a glass woven fiber layer at top and bottom (GPG) demonstrate 291.43% higher load resistance (peak force) compared to PALF composite (P). Also, this study shows that a positive hybrid effect of PALF as a reinforcement was seen where the GPG shows the time delay for initiating damage and propagating it through the whole sample compared to GGGG. Besides that, the presence of woven glass fiber layer in GPG helps change the composite characteristic from less elastic properties to more elastic properties, and the damage could be seen visually.

## Figures and Tables

**Table 1:** Fiber composition.

Composite	PALF (P)	Glass Fiber Woven Mat (G)	Layup Sequence
PALF/Epoxy	10%wt	-	P
Hybrid	5%wt	2 layer	GPG
Synthetic	-	4 layer	GGGG

**Table 2:** Impact energy range for the low-velocity impact test.

Sample	Impact Energy, J
P	1.0
	2.0
GPG	2.0
	3.0
	6.0
	9.0
GGGG	9.0
	10.5
	12.0

**Table 3:** Natural fiber and its hybrid composite damage associated with LVI.

Fiber	Polymer	Damage Mode (Visual)		Reference
		Front	Back	
Bamboo powder fibers	epoxy	Matrix crack		[15]
Bamboo powder fibers + Glass fiber mat	epoxy	Localised delamination	Bulge with crack	[15]
UD PALF	Polylactic acid (PLA)	Circular dent with crack	Bulge with crack	[32]
UD Sisal	epoxy	Single long line crack		[33]
Kenaf fiber mat	epoxy	Crack along the horizontal (major) and vertical (minor)		[27]
Kenaf fiber mat + glass fiber mat	epoxy	Localised delamination with crack along the horizontal (major) and vertical (minor)		[27]
Kenaf fiber mat + glass fiber mat	epoxy	Delamination at the center	-	[35]
UD Kenaf laminates	epoxy	-	Cross-shaped bulge with fiber breakage	[36]

## Data Availability

The data presented in this study are available on request from the corresponding author.
